# Profound Vitamin D Deficiency in a Diverse Group of Women during Pregnancy Living in a Sun-Rich Environment at Latitude 32°N

**DOI:** 10.1155/2010/917428

**Published:** 2010-12-09

**Authors:** Stuart A. Hamilton, Rebecca McNeil, Bruce W. Hollis, Deborah J. Davis, Joyce Winkler, Carolina Cook, Gloria Warner, Betty Bivens, Patrick McShane, Carol L. Wagner

**Affiliations:** ^1^The Eau Claire Study Group, Department of Obstetrics and Gynecology, Eau Claire Cooperative Health Centers, Columbia, SC 29204, USA; ^2^Durham Epidemiologic Research and Information Center, Durham Veterans Affairs Medical Center, 508 Fulton Street, Durham, NC 27705, USA; ^3^Division of Neonatology, Department of Pediatrics, Medical University of SC, 173 Ashley Avenue, MSC 513, Charleston, SC 29425, USA

## Abstract

*Objective*: Determine prevalence of vitamin D deficiency (VDD) in a diverse group of women presenting for obstetrical care at two community health centers in South Carolina at latitude 32°N. *Methods and Design*: Any pregnant woman presenting for care at 2 community health centers was eligible to participate. Sociodemographic and clinical history were recorded. A single blood sample was taken to measure circulating 25(OH)D as indicator of vitamin D status [25(OH)D < 20 ng/mL (50 nmol/L deficiency; <32 ng/mL (80 nmol/L) insufficiency]. Total serum calcium, phosphorus, creatinine, and intact parathyroid hormone also were measured. *Results*: 559 women, [mean age 25.0 ± 5.4 (range 14–43) years] participated: African American (48%), Hispanic (38%), Caucasian/Other (14%). Mean gestational age was 18.5 ± 8.4 (median 14.6, range 6.4–39.6) weeks' gestation. 48% were VDD; an additional 37% insufficient. Greatest degree was in the African American women (68% deficient; 94% insufficient). In multivariable regression, 25(OH)D retained a significant negative association with PTH (*P* < .001). *Conclusions*: VDD was high in a diverse group of women, greatest in those of darker pigmentation. The negative correlation between 25(OH)D and PTH confirms their corroborative use as biomarkers of VDD. These findings raise the issue of adequacy of current vitamin D recommendations for pregnant women.

## 1. Introduction

Much has happened in the world of vitamin D research since the 2002 Centers for Disease Control report of the widespread deficiency in African-American women in their childbearing years [1]. Vitamin D is no longer seen as just a “childhood” vitamin that has little bearing on health beyond bone and calcium metabolism [2–4]. Rather, vitamin D is now seen as a preprohormone whose active metabolites act not only to ensure calcium homeostasis and bone health [5] but also act as mediators of immune function in general [6, 7], and specific to pregnancy, play a role in immune tolerance, insulin resistance, and preeclampsia [8–12]. Studies to support this premise have increased significantly in number during the past five years [13–30], and with frequent lay press articles and featured news items about vitamin D, public understanding of vitamin D's importance in maintaining health has increased. Yet, even with this understanding, the translation of this knowledge into clinical practice in the community is still minimal. Much of the work concerning pregnant women has been performed at university-based clinics, whose relevance to community health centers is often in question. Earlier reports of vitamin D deficiency during pregnancy have focused on women living in northern Europe (latitude 50–55°N), which did not include women of diverse racial/ethnic backgrounds living in a sun-rich environment [5, 31, 32]. Theoretically, women at lower latitudes, despite darker pigmentation, should have less vitamin D deficiency because of the greater likelihood of effective sunlight exposure to induce endogenous vitamin D synthesis [33].

As a result of the concern regarding the prevalence of vitamin D deficiency among pregnant women receiving care at community health centers, and to clarify earlier scientific studies which reported that a majority of African-American and Hispanic women are vitamin D deficient [34–40], a cross-sectional study of pregnant women presenting at a community health center network in South Carolina at latitude 32°N was designed and implemented. The aim of this study was to define the prevalence of vitamin D deficiency in a large and ethnically diverse cross-sectional sample of pregnant women living in a sun-rich environment with ample access to sunlight during most of the year. It was hypothesized that despite South Carolina's lower latitude and abundance of sunlight, given modern-day lifestyles that often preclude sunlight exposure, the majority of the women presenting for obstetrical care at this community health center network would have vitamin D insufficiency and a significant number would have outright vitamin D deficiency.

## 2. Methods and Study Design

### 2.1. Subjects

The study was approved by MUSC's Institutional Review Board for Human Research (HR# 16476) and the Palmetto Baptist Hospital Institutional Review Board for Human Research (PH IRB# 2007-25). The only inclusion criteria were age of 14 years or more, and confirmed pregnancy at the time of the clinic visit to one of two urban community health networks in Columbia and Charleston, SC. There were no exclusion criteria for participation in the study. This study was conducted from November 21, 2006 through October 31, 2008.

### 2.2. Procedure

After providing written informed consent, women were asked a series of questions about their sociodemographic characteristics, including self-described race/ethnicity and health status. Sociodemographic and clinical characteristics and obstetrical history were recorded for each woman on a standardized data form. Each woman had been prescribed a prenatal vitamin containing 400 IU of vitamin D at her first prenatal visit; compliance with the prenatal vitamin regimen at the time of the blood sampling was also recorded. A blood sample was drawn with the gestational age (weeks) recorded at the time of the blood sampling. Blood was processed and serum separated for later analysis for total circulating 25-hydroxy-vitamin D [25(OH)D], serum calcium, phosphorus, and creatinine, and intact parathyroid hormone (PTH). Season was calculated from the date that the blood sample was drawn according to the following categories: winter, December-March; spring, April-May; summer, June-September; fall, October-November.

### 2.3. Sociodemographic Characteristics of the Cohort

Sociodemographic data were collected on each subject using a standardized data entry form. Specifically, information about maternal age, self-identified ethnicity, health insurance status, marital status, employment status, and educational status was collected. Given the putative relationship between cigarette smoking and PTH [41, 42], information also was collected about cigarette smoking during the current pregnancy.

### 2.4. Clinical Characteristics of the Cohort

Clinical characteristics included gravidity, parity, and history of preterm birth, preeclampsia, and gestational diabetes during prior pregnancies. Information regarding maternal history of diabetes (type 1 or 2) and chronic hypertension was also recorded. The body mass index (or BMI; kg/m^2^) was calculated for each woman based on her prepregnancy weight. Women with a prepregnancy BMI equal to or greater than 30 were considered obese.

### 2.5. Degree of Skin Pigmentation

The degree of skin pigmentation at each of four body sites (forehead, inner underarm, forearm and knee) was recorded using the Smart Probe 400 (IMS, Inc., Milford, CT) and a skin tone chart (IMS, Inc.) on a continuous scale from 0–100, where absolute black is 0 and absolute white is 100.

### 2.6. Measurement of 25(OH)D

Total circulating 25(OH)D concentrations (ng/mL) were measured using a commercially available radioimmunoassay (RIA) performed in the laboratory of Dr. Bruce Hollis (Diasorin, Stillwater, MN). This laboratory participated throughout the study in the DEQAS quarterly quality control program for vitamin D. *A priori*, “deficiency” was defined as a concentration <20 ng/mL (<50 nmol/L) and “insufficiency” as a concentration between 20 and 32 ng/mL (50–80 nmol/L) [43].

### 2.7. Measurement of Parathyroid Hormone (PTH)

Intact parathyroid hormone (pg/mL) was measured by a commercially available immunoradiometric assay (N-Tact PTH IRMA; Diasorin, Stillwater, MN) following the manufacturer's guidelines.

### 2.8. Statistical Analysis

Continuous and ordinal demographic and clinical characteristics were reported as mean ± SD and median (range). Categorical variables were reported as frequency and percent. Characteristics were tabulated for the entire cohort and according to self-reported race/ethnicity (African-American, Hispanic, Caucasian/Other).

The primary endpoint for this study was total circulating 25(OH)D (ng/mL). Secondary endpoints included serum PTH (pg/mL), calcium (mg/dL), creatinine (mg/dL), and phosphorus (mg/dL). The associations between these continuous endpoints and sociodemographic and clinical characteristics were examined using multivariable linear regression, loess regression, and the Kruskal-Wallis test. Participants were categorized as vitamin D deficient, insufficient, or sufficient as described above; the associations between vitamin D category and predictive factors were evaluated using the chi-square test or Fisher's exact test (categorical predictors) and logistic regression (continuous predictors and/or multivariable models). Results were considered statistically significant for *P*-values less than  .05. All analyses were performed using SAS Version 9.1 (SAS Institute Inc., Cary, NC).

## 3. Results

As shown in Table 1, 559 women recruited from two community health center networks in Columbia and Charleston, SC at latitude 32°N participated in the study; 4 participants enrolled twice due to repeated pregnancies during the enrollment period. The mean age was 25.0 ± 5.4 (range 14–43) years. The self-reported ethnicities of the participants were African-American (48.1%), Hispanic (38.1%), Caucasian (9.5%) and Other (Asian and American Indian) (4.3%). No participants younger than age 17 reported having a high school diploma or higher education. The earliest age reported for each category of education was high school, 17 years; some college, 18 years; associate degree or higher, 19 years. Of the participants aged 17 years or greater, 73.3% reported at least a high school diploma. Of those aged 18 years or greater, 34.8% reported at least some college. Of those age 19 years or greater, 9.8% reported achieving an associate's degree or higher (*P* < .001 for association with race/ethnicity). Women in this cohort were more likely to have no insurance (36.3%) or Medicaid (44.7%) than to have private insurance (*P* < .001 for association with race). The majority of the participants (55.1%) were enrolled during the summer months (June–September).

A total of 252 women (45.1%) were employed at the time of the study, working a median number of 39 hours worked per week (range 0–84). A total of 88 (15.7%) reported having stopped their employment due to pregnancy. In addition, 44 (7.9%) of the study participants were students at the time of enrollment into the study. 

A total of 557 of the 559 participants completed the clinical characteristics survey. For the majority of women in the cohort, pregnancy was unplanned (64.9%, *P* < .001 for association with race). The overall median self-rated health status of the women at the time of enrollment was 9 (excellent), and ranged from 3–10 (mean ± SD, 8.8 ± 1.2). A total of 190 (34%) were primigravid women. The median number of prior pregnancies in the cohort was 1. Of the 367 women with a stated history of a prior pregnancy, 159 (28.4% total cohort) had 1 prior pregnancy, 97 (17.4% total cohort) had 2 prior pregnancies, and 111 (19.9% total cohort) had ≥3 prior pregnancies. Of those women with a history of one or more prior pregnancies, 41 (7.3% total cohort) had a history of preterm birth. In addition, preeclampsia was reported in a prior pregnancy in 21 (3.8%) women, and gestational diabetes was reported in 15 (2.7%) women. A small percentage (5.2%) of the women reported dietary restrictions that may have influenced their vitamin D status; 3% reported such restrictions for health reasons such as type 2 diabetes, 0.9% for religious reasons (vegetarian), and 1.3% gave no reason. 

**Figure 1:**
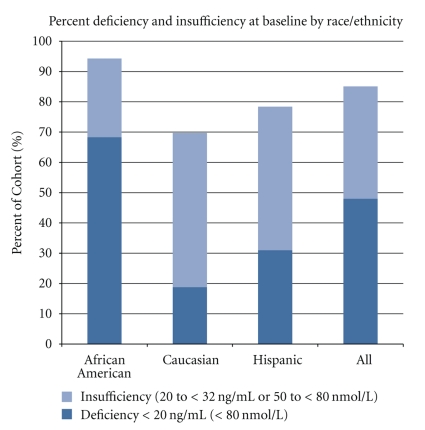
Baseline circulating 25(OH)D levels (ng/mL).

The mean vitamin D status of the cohort, as measured by total circulating 25(OH)D, was 21.7 ± 9.7 ng/mL (range 3.8 to 73.8; unavailable for 7 participants). Overall, 48% of the participants were vitamin D deficient, with an additional 37.1% insufficient. As shown in Figure 1, this varied significantly by self-identified race/ethnicity (*P* < .001). The greatest degrees of deficiency and insufficiency were seen in the African-American women, with 68.3% having frank deficiency (concentration <20 ng/mL) and 94.3% having either deficiency or insufficiency (concentration <32 ng/mL).  Table 2 provides additional details regarding the prevalence of the degrees of deficiency by race/ethnicity. In univariate logistic regression models, African-American women had approximately 8 times the odds of vitamin D deficiency of Hispanic women, and 20 times the odds of vitamin D deficiency of Caucasian women (*P* < .001 for both comparisons). Vitamin D status as measured by 25(OH)D was also analyzed by season: after controlling for race, there was no significant evidence of seasonal variation in the prevalence of vitamin D insufficiency and/or deficiency in this population.

The mean gestational age was 18.5 ± 8.4 (median 14.6, range 6.4–39.6) weeks of gestation (see Table 1). As shown in Table 5, a total of 56.0% of the cohort were less than 16 weeks of gestation, 24.7% were between 16 and 28 weeks of gestation, and 17.2% were at 28 weeks or more of gestation (0.9% did not report a gestational age). The odds of vitamin D deficiency (as measured by 25(OH)D concentration) did not appear to change as a function of gestational age; women in their later stages of pregnancy had a similar odds of vitamin D deficiency and insufficiency for a given race/ethnicity than women who had their 25(OH)D concentrations measured earlier in pregnancy (*P* = .43). A total of 58 (10.4%) participants reported using prenatal vitamins or multivitamins; this was weakly associated with race (*P* = .065). After controlling for race, prenatal/multivitamin use (containing 400 IU vitamin D/day) was not associated with 25(OH)D concentration (*P* = .11).

We evaluated the use of skin pigmentation score as a surrogate for race; this resulted in a strong correlation between 25(OH)D concentration and pigmentation score (*P* < .002 for all measurement sites). As expected, this association became nonsignificant when race was added to the model. A related measure of sun exposure, derived as the difference in pigmentation scores between the inner underarm and forearm, was not associated with 25(OH)D concentration (*P* = .27), as both values changed pursuant to sun exposure. 

Multinomial logistic regression models for the three vitamin D categories (deficient, insufficient, and sufficient) were constructed to evaluate the association between vitamin D status and age, gravidity, race, and season of measurement. The results of these univariate models are described in Table 3. Specifically, obesity and race demonstrated a significant association with vitamin D category, and obesity retained a significant relationship with vitamin D category after controlling for race (*P* = .01). 

The association between relevant prior and current medical conditions and present vitamin D status is summarized in Table 4. In exploratory univariate analyses, vitamin D concentrations differed significantly or nearly significantly between subgroups based on hypertension (*P* = .055), diabetes (*P* = .023), varicose veins (*P* = .056), and the presence of any STD (*P* < .001). After controlling for race, 25(OH)D concentration differed marginally between subgroups defined by the presence/history of psychiatric illness (*P* = .058) and diabetes (*P* = .074). In all of the models described in Table 3, race was a highly significant independent predictor of 25(OH)D concentration. 

**Figure 2:**
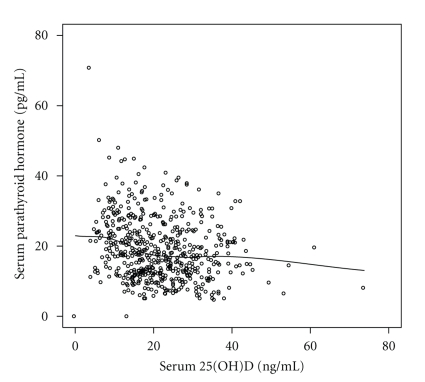
Scatterplot of PTH and 25(OH)D concentrations. The overlaid line modeling the relationship between PTH and 25(OH)D was fit using loess regression (locally weighted smoothed regression).

Correlation analysis found that 25(OH)D concentration was weakly, but significantly, associated with serum calcium (*r* = 0.12, *P* = .007) and negatively associated with PTH (*r* = −0.24, *P* < .001; see Figure 2) and marginally associated with serum creatinine (*r* = −0.08, *P* = .055). Fitting of a three-parameter exponential decay model suggested that the inflection point at which 25(OH)D and the effect of higher 25(OH)D had no further effect on PTH, that is, the relationship reached a plateau was approximately 16.7 ng/mL (95% CI 14.8–18.6 ng/mL). There was no evidence of association between 25(OH)D and serum phosphorus (*r* = 0.02, *P* = .70). Controlling for race did not change the association between 25(OH)D and calcium. In a multivariable model containing both race and 25(OH)D, race was a significant independent predictor of serum creatinine (*P* < .001), and the association between creatinine and 25(OH)D after controlling for race was barely significant (*P* = .047). Neither race nor 25(OH)D was associated with serum phosphorus in a multivariable model. 

The overall mean ± SD PTH concentration was 19.2 ± 8.8 (range 4.7–70.8) pg/mL. As expected, PTH demonstrated a positive association with gestational age category (see Table 5; *P* < .001). There was no evidence of association between PTH and race (*P* = .51; see Table 6(a)). As shown in Table 6(b), in a model of PTH, race did not add significantly to the model (*P* = .66). Similarly, there was no significant interaction between race and gestational age (*P* = .23), suggesting that there was no detectable difference between races in the association between PTH and gestational age. These findings suggest that the positive association between PTH and gestational age was consistent among racial/ethnic categories. 

After accounting for the effect of gestational age, 25(OH)D retained a significant negative association with PTH (*P* < .001). In addition, there was a trend toward increased magnitude of association between PTH and gestational age among participants with lower 25(OH)D (*P* = .11 for interaction), suggesting that the increase in PTH due to endogenous production by the placenta is moderated by 25(OH)D across the full range of gestational ages. PTH was negatively associated with serum calcium (*r* = −0.38, *P* < .001) even after controlling for gestational age, and weakly associated with serum phosphorus (*r* = −0.11, *P* = .011); calcium and phosphorus were also associated (*r* = 0.25, *P* < .001). 

With regard to cigarette smoking, there were 35 women in the cohort who reported use of cigarettes during the current pregnancy (6.3%). PTH was significantly associated with reported smoking status (nonsmokers 19.4 pg/mL, smokers 16.6 pg/mL, *P* = .0387). The association between PTH and gestational age category, however, was unchanged in effect estimates or significance after controlling for smoking.

## 4. Discussion

Vitamin D deficiency in women of diverse ethnic background, including those of African-American and Hispanic descent, was prevalent in this cohort, more prevalent than what has been reported earlier in women of those ethnicities living at higher latitudes. Overall, 48% of the participants were vitamin D deficient, with an additional 37% being insufficient. This degree of deficiency was noted despite that these women lived in a sun-rich climate for most of the year. Women who reported taking a prenatal vitamin containing 400 IU vitamin D did not differ from those who did not in vitamin D status as measured by 25(OH)D. 

While present in Caucasian women, the greatest prevalence of deficiency was noted in those women of darker pigmentation, specifically, African-American and Hispanic women. While there was a significant variation in 25(OH)D concentrations between racial/ethnic categories, the deficiency did not appear to change as a function of gestational age; women in their later stages of pregnancy had a similar risk of vitamin D deficiency and insufficiency for a given race/ethnicity compared with women who had their 25(OH)D concentrations measured earlier in pregnancy. This suggests that any reductions in sun exposure attributable to the limited mobility or lower maternal body self-image experienced during later stages of pregnancy did not have a meaningful impact on 25(OH)D concentrations. While we lack specific sun exposure data, we hypothesize that this apparent absence of association may be due to minimal sun exposure among our participants at all gestational ages, as reflected by their low 25(OH)D status. 

Prior studies have reported conflicting data about PTH concentrations during pregnancy. Some investigators reported that PTH bioactivity during pregnancy is either in the normal range [44–47] or increases [48–52];while others have reported that intact PTH concentration is approximately half that seen in the nonpregnant woman [53, 54]. Okonofua et al. [55], in response to these differences in study findings, designed a study to elucidate PTH activity during pregnancy. In their study of Asian and Caucasian women in the UK, PTH concentrations were greater in Asian than Caucasian women, but despite these differences, both groups maintained blood calcium concentrations in the normal range. When both groups were combined, PTH was inversely related to both plasma calcium and 25(OH)D. In contrast, when the groups were analyzed separately, the inverse correlation between PTH and calcium persisted whereas the relationship between PTH and 25(OH)D disappeared. This observation was the first of its kind in the literature. The authors suggested that this significant inverse relationship between calcium and PTH concentrations in a group of women whose calcium concentrations were within the normal range was consistent with the premise that PTH secretion is responsive to alternations in calcium concentration during pregnancy irrespective of the underlying trends toward an increase in PTH during pregnancy. 

In our study, while PTH demonstrated a positive association with gestational age, there was no evidence of an interaction between gestational age and race, which suggests that the positive association between PTH and gestational age was consistent among racial/ethnic categories. After accounting for the effect of gestational age through multivariable regression, 25(OH)D status retained a significant negative association with PTH, confirming that even during pregnancy, PTH is a predictor of vitamin D deficiency. Our results regarding the association between PTH and calcium were consistent with those of Okonofua et al. [55], from two decades ago with a population of women of diverse ethnicity.

Using both circulating 25(OH)D and PTH as markers of vitamin D deficiency, these findings underscore recent reports of widespread vitamin D deficiency among pregnant women, even in areas of the world with ample sunlight. This is concerning in that the effects of vitamin D deficiency among pregnancy women are not limited to the women: if a mother has vitamin D insufficiency, or worse, deficiency, her fetus is developing in a hypovitaminotic state. At its most basic concentration, such a burden of deficiency sets the stage of subsequent deficiency for the neonate and infant whose stores of vitamin D were set during pregnancy. Infants who are vitamin D deficient at birth are at the highest risk of impaired bone development [5] that appears to persist in even later childhood [37]. What is most concerning is that use of prenatal vitamins containing 400 IU/tablet was irrelevant in affecting vitamin D status: those who took their prescribed prenatal vitamin containing vitamin D did not differ from those who did not. Women taking their prenatal vitamins should be counseled that the amount of vitamin D contained in their prenatal vitamin will not affect their vitamin D status [33, 56, 57]. 

The strengths of the study are that a large and diverse group of women at various stages of pregnancy were studied to: (1) ascertain their vitamin D status at the time of presentation to a community health center and (2) find what factors were most predictive of that status in a sun-rich environment where theoretically there are no limitations to achieving an adequate vitamin D status. Potential limitations of this study include that the active form of vitamin D, 1,25(OH)_2_D, was not measured. In addition, while maternal use of prenatal vitamins was recorded, compliance with this regimen was not ascertained through pill counts. We have established previously that pill counts are not necessarily predictive of adherence to protocol and only biochemical measures give one a true indication of adherence [58].

Given recent discoveries that link vitamin D with the innate immune system [6], it is not unreasonable to predict that deficiencies during fetal development could have lasting sequelae on the child, not only in terms of bone mineralization, but also in terms of immune development that becomes the basis for later derangements seen with long-latency diseases such as multiple sclerosis, rheumatoid arthritis, diabetes, and certain cancers [59]. However, little is currently known about the long-term sequelae of fetal hypovitaminosis D on immune function. Studies to examine the benefit of higher doses of vitamin D during pregnancy suggest that the dose of vitamin D found in most prenatal vitamins—400 IU—is inadequate to meet the needs of the pregnant woman and her growing fetus [11, 32, 34, 39, 60–67]. Taking into account vitamin D's emerging role in immune maintenance throughout the body and the mounting evidence to support the importance of vitamin D in maintaining good health, at the very least, women who are deficient in vitamin D should be counseled regarding the risks of vitamin D deficiency for themselves and their offspring and recommended a therapy to ensure vitamin D adequacy [60–62, 68–70].

## Figures and Tables

**Table 1:** Sociodemographic and clinical characteristics at time of enrollment, by self-reported race/ethnicity.

*Sociodemographic and clinical characteristics*	*Total cohort* *N* = 559	*African-American* *N* = 269	*Hispanic* *N* = 213	*Caucasian* *N* = 53	*Other ethnicities* *N* = 24	*P*-*value *
Maternal age* (Mean ± SD)	25.0 ± 5.4	23.9 ± 5.1	26.1 ± 5.4	25.8 ± 5.7	26.8 ± 5.7	<.001
Gestational age* (Mean ± SD)	18.5 ± 8.4	18.0 ± 8.0	19.2 ± 9.0	17.7 ± 7.8	19.3 ± 8.2	.49

*Highest education achieved*						

<High school	155 (28%)	43 (16%)	102 (48%)	8 (15%)	2 (8%)	
High school	215 (38%)	100 (37%)	90 (42%)	18 (34%)	7 (29%)	
Some college	138 (25%)	101 (38%)	15 (7%)	16 (30%)	6 (25%)	<.001
Assoc. degree or higher	51 (9%)	25 (9%)	6 (3%)	11 (21%)	9 (38%)	
Employment (yes)	252 (45%)	149 (55%)	68 (32%)	28 (53%)	7 (29%)	<.001

*Insurance status*						

None	203 (36%)	4 (1%)	189 (89%)	3 (6%)	7 (29%)	
Medicaid	250 (45%)	207 (77%)	12 (6%)	23 (43%)	8 (33%)	<.001
Private	106 (19%)	58 (22%)	12 (6%)	27 (51%)	9 (38%)	

*Subjective health rating scale*						

(Median, range)**	9 3–10	9 3–10	9 3–10	9 7–10	10 6–10	.040

*Prior obstetrical history*						

Preterm birth	41 (7.3%)	27 (10.0%)	11 (5.2%)	2 (3.8%)	1 (4.2%)	.17
Preeclampsia	21 (3.8%)	13 (4.8%)	5 (2.4%)	2 (3.8%)	1 (4.2%)	.48
Gestational diabetes	15 (2.7%)	9 (3.4%)	3 (1.4%)	2 (3.8%)	1 (4.2%)	.30
Diabetes Mellitus (type 1 or 2)	8 (1.4%)	6 (2.2%)	2 (0.9%)	0	0	.64
Chronic hypertension	21 (3.8%)	15 (15.6%)	1 (1.9%)	3 (1.4%)	2 (8.3%)	.036
Planned pregnancy	196 (35%)	43 (16%)	121 (57%)	21 (40%)	11 (46%)	<.001
Primigravida^†^	190 (34.1%)	98 (36.4%)	65 (30.7%)	19 (36.5%)	8 (33.3%)	.59
BMI ≥ 30^†^	174 (31.1%)	94 (34.9%)	65 (30%)	11 (21%)	4 (17%)	.076

*Season* ^†^						

April-May	79 (14.1%)	45 (16.7%)	23 (10.8%)	7 (13.2%)	4 (16.7%)	<.0001
June–September	309 (55.3%)	127 (47.2%)	138 (64.8%)	27 (50.9%)	17 (70.8%)	
October-November	79 (14.1%)	32 (11.9%)	38 (17.8%)	8 (15.1%)	1 (4.2%)	
December–March	92 (16.5%)	65 (24.2%)	14 (6.6%)	11 (20.8%)	2 (8.3%)	

*P*-values refer to the comparison between the four racial/ethnic categories reported.

*ANOVA; ^†^Chi-square; **Subjective Health Rating Scale 0 = poor to 10 = excellent.

**Table 2:** Prevalence of vitamin D deficiency (*N*, %) according to race/ethnicity.

Deficiency category	Total cohort *N* = 552	African-American *N* = 262	Hispanic *N* = 213	Caucasian *N* = 53	Other Ethnicities *N* = 24
<12 ng/mL	87 (15.8%)	67 (25.6%)	13 (6.1%)	4 (7.5%)	3 (12.5%)
12–19 ng/mL	178 (32.2%)	112 (42.7%)	53 (24.9%)	6 (11.3%)	7 (29.2%)
20–31 ng/mL	205 (37.1%)	68 (26.0%)	101 (47.4%)	27 (50.9%)	10 (41.7%)
32+ ng/mL	82 (14.9%)	15 (5.7%)	46 (21.6%)	16 (30.0%)	4 (16.7%)

**Table 3:** Multinomial logistic regression model for the univariate associations between vitamin D concentration and age, gravidity, BMI, race, and season of measurement.

Variable	Vitamin D group	Odds ratio	95% CI	*P*-value
Age < 25	<20	1.35	0.82–2.21	.24
	20–31	1.28	0.77–2.14	.35
	32+	1.00 (ref)	—	—

Primigravida	<20	0.82	0.49–1.38	.45
	20–31	0.99	0.58–1.71	.99
	32+	1.00 (ref)	—	—

Obesity (BMI ≥ 30)	<20	2.19	1.23–3.90	.008
	20–31	1.21	0.66–2.22	.54
	32+	1.00 (ref)	—	—

African-American (versus Caucasian)	<20	20.28	7.91–52.02	<.0001
	20–31	2.96	1.29–6.78	.007
	32+	1.00 (ref)	—	—

Hispanic (versus Caucasian)	<20	2.44	1.03–5.81	.046
	20–31	1.44	0.71–2.90	.51
	32+	1.00 (ref)	—	—

Summer Months	<20	0.60	0.34–1.05	.073
	20–31	0.88	0.49–1.59	.67
	32+	1.00 (ref)	—	—

Odds ratios are univariate (unadjusted for the other listed covariates); no additional covariates were included in the model. Vitamin D group sample sizes are as follows: <20, *n* = 265; 20–31, *n* = 205; 32+, *n* = 82. Missing data were as follows: gravidity (*n* = 2). The race category of “Other” was excluded from this analysis due to small sample size.

**Table 4:** History of prior or current medical conditions of study participants and 25(OH)D concentrations at time of enrollment.

Medical condition	*N* (%) with history	25(OH)D Concentration of those *with history *	25(OH)D Concentration of those *without history *	*P*-value
Urinary tract infection	160 (28.6%)	20.7 (8.9)	21.7 (10.3)	.27
Abnormal PAP smear	92 (16.5%)	21.0 (11.5)	21.5 (9.6)	.62
History of preterm labor or miscarriage	41 (7.3%)	18.3 (11.2)	21.7 (9.8)	.039*
Anemia	47 (8.4%)	19.5 (7.1)	21.6 (10.2)	.16
Asthma	45 (8.1%)	19.6 (8.3)	21.6 (10.1)	.21
Psychiatric illness	36 (6.4%)	19.5 (8.1)	21.6 (10.1)	.23
Bipolar disorder	5 (0.9%)	23.2 (10.2)	21.4 (10.0)	.68
Depression	29 (5.2%)	19.5 (8.1)	21.5 (10.0)	.29
Varicose veins	23 (4.1%)	25.3 (13.4)	21.3 (9.8)	.056
Hypertension	21 (3.8%)	17.3 (10.9)	21.6 (9.9)	.055
Kidney stones and/or disease	10 (1.8%)	21.7 (6.4)	21.4 (10.0)	.93
Diabetes	8 (1.4%)	13.5 (4.5)	21.5 (10.0)	.023*
Seizures	7 (1.3%)	19.5 (8.0)	21.5 (10.0)	.61
Tuberculosis	6 (1.1%)	22.4 (5.8)	21.4 (10.0)	.81
Thyroid disease	5 (0.9%)	19.8 (11.5)	21.4 (10.0)	.71
Any Reported Sexually Transmitted Disease	126 (22.5%)	18.3 (9.4)	22.3 (9.9)	<.001*
Chlamydia	99 (17.7%)	18.2 (9.8)	22.1 (9.9)	.0003*
Gonorrhea	29 (5.2%)	19.6 (7.8)	21.5 (10.1)	.32
Genital herpes	11 (2.0%)	18.1 (9.7)	21.5 (10.0)	.26
Genital warts	10 (1.8%)	19.2 (5.7)	21.5 (10.0)	.48

**Table 5:** Serum 25(OH)D and PTH according to gestational age.

	<15 6/7 weeks (*n* = 313)	16–27 6/7 weeks (*n* = 138)	28+ weeks (*n* = 96)	*P*-value
25(OH)D (ng/mL) Mean ± SD	21.9 ± 9.7	21.2 ± 9.1	22.2 ± 10.7	.83
Median (range)	20.5 (4.2–73.8)	20.4 (5.1–44.1)	20.4 (3.8–61.3)	
PTH (pg/mL)* Mean ± SD	16.9 ± 7.2	21.3 ± 9.0	23.9 ± 10.6	<.001
Median (range)	15.7 (4.7–39.5)	20.2 (5.8–48.0)	22.4 (6.6–70.8)	

Reported as mean (SD) in row 1, and median (range) in row 2. *Missing data (*n* = 1) for PTH.

**Table:** (a) Estimated PTH (pg/mL) levels for each category of self-identified race and ethnicity^†^

	N*	Mean (SD)	Range
All participants	551	19.2 (8.8)	4.7–70.8
African-American	261	19.6 (9.5)	5.1–70.8
Caucasian	53	17.9 (9.1)	6.2–38.7
Hispanic	213	19.1 (8.0)	4.7–40.9
Other	24	18.0 (6.6)	7.6–32.6

^†^Eight subjects had insufficient blood sample to measure PTH and thus, were excluded from this table.

**Table:** (b) Serum PTH (pg/mL) according to gestational age and race/ethnicity**

	PTH According to Gestational Age and Race/Ethnicity		
	<32 weeks (*n* = 484)	32–35 weeks (*n* = 36)	36+ weeks (*n* = 26)
All participants	18.7 (8.5) 17.4 (4.7–70.8)	21.5 (9.1) 22.1 (6.6–44.2)	26.9 (9.6) 27.9 (9.6–45.2)
African-American	19.0 (9.0) 17.3 (5.1–70.8)	22.0 (10.9) 22.8 (6.6–44.2)	32.9 (9.8) 33.2 (17.2–45.2)
Caucasian	17.6 (8.8) 15.3 (6.4–38.7)	20.3 (9.5) 21.7 (10.2–29.1)	27.8 (—) 27.8 (18.2–37.3)
Hispanic	18.5 (8.0) 17.7 (4.7–40.9)	22.6 (7.0) 24.2 (7.0–30.8)	23.1 (7.5) 21.8 (9.6–33.6)
Other	18.5 (6.5) 16.9 (10.7–32.6)	11.8 (—) 11.8 (7.6–16.0)	

**Reported as mean (SD) in row 1 and median (range) in row 2. Blank cells are those that contain no participants. SD was not estimated in cells that contain fewer than 3 observations.
